# Enteric parasitic infections in children and dogs in resource-poor communities in northeastern Brazil: Identifying priority prevention and control areas

**DOI:** 10.1371/journal.pntd.0008378

**Published:** 2020-06-09

**Authors:** Tatiani Vitor Harvey, Alice M. Tang, Anaiá da Paixao Sevá, Camila Albano dos Santos, Silvia Maria Santos Carvalho, Christiane Maria Barcellos Magalhães da Rocha, Bruno César Miranda Oliveira, George Rego Albuquerque

**Affiliations:** 1 Departamento de Ciências Agrárias e Ambientais, Programa de Pós-Graduação em Ciência Animal, Universidade Estadual de Santa Cruz—UESC, Ilhéus, Bahia, Brasil; 2 Department of Public Health & Community Medicine, School of Medicine, Tufts University, Boston, Massachusetts, United States of América; 3 Departamento de Medicina Preventiva e Saúde Animal, Faculdade de Medicina Veterinária e Zootecnia, Universidade de São Paulo–USP, São Paulo, São Paulo, Brasil; 4 Departamento de Ciências Agrárias e Ambientais, Faculdade de Medicina Veterinária, Universidade Estadual de Santa Cruz—UESC, Ilhéus, Bahia, Brasil; 5 Departamento de Ciências Biológicas, Universidade Estadual de Santa Cruz -UESC, Ilhéus, Bahia, Brasil; 6 Departamento de Medicina Veterinária, Universidade Federal de Lavras—UFLA, Lavras, Minas Gerais, Brasil; 7 Departamento de Apoio, Produção e Saúde Animal, Universidade Estadual Paulista, Faculdade de Medicina Veterinária, Araçatuba, SP, Brasil; University of Queensland School of Veterinary Science, AUSTRALIA

## Abstract

The aim of this study was to determine the prevalence and risk factors of the main enteric parasitic infections that affect children and dogs in the municipality of Ilhéus, Bahia, Brazil; and to identify the geopolitical areas that should receive priority interventions to combat them. Between March and November 2016, fecal samples of 143 dogs and 193 children aged 1 month to 5 years were collected in 40 rural and semirural communities using a systematic sampling approach, stratified by district. Samples were collected by legal guardians of the children and / or dog owners. Eggs, larvae, cysts and oocysts of parasites were concentrated by centrifugal-flotation and centrifugal-sedimentation, and acid-resistant staining was used to visualize parasites. One hundred and thirty-two children (68.4%), 111 dogs (77.6%) and 199 (73.7%) dog fecal samples collected from streets were parasitized. Giardiasis, cryptosporidiosis, amoeba infections and hookworm were the most frequent infections in all studied populations, in addition to trichuriasis in dogs and ascaridiasis in children. A predominance of *Giardia* and hookworms was observed in children and dogs, respectively. The coastal districts of Aritaguá, Olivença and the main district had a higher parasitic diversity and overlapping of important potential zoonotic infections. Age over one year (p<0.001), adjusted OR = 3.65; 95% CI = 1.86–7.16) and income below the minimum monthly salary (p = 0.02, adjusted OR = 2.78, 95% CI = 1.17–6.59) were the main factors associated with intestinal parasitic infections in children and dogs, respectively. The coastal districts of Aritaguá and Olivença and the main district should be prioritized through enteric disease control programs, and the factors associated with infections must be considered in the design of health interventions in these districts. The integration between affirmative income actions and investments to improve the health infrastructure of these communities may work more effectively than current preventive measures to combat enteric parasites.

## Author summary

The maintenance of high prevalence of enteric parasitic diseases in human and animal populations is an indisputable fact in the current scientific context. Despite all the advances in ecology, pathology, epidemiology and treatment, the control of these diseases remains ineffective and is a major problem in public health management. New control strategies should be tested in order to optimize the actions of current preventive programs and thus reduce prevalence. These strategies should include identification and mapping of geopolitical areas experiencing a greater frequency and overlap of enteric parasitic infections, and they must include the prevalence in both canine and human populations. This study indicates that poverty is strongly associated with the parasite's frequency. In a One Health approach, it demonstrates that affirmative actions of citizenship and income can be preventive health measures, and it also demonstrates the areas in which deworming actions must be a priority through control actions based on risk.

## Introduction

Knowledge of the distribution of enteric parasitic diseases, as well as the areas of overlap, is critical for identifying hotspots where there is a need for integrated prevention and control interventions [1]. These actions are particularly important in underdeveloped areas, where social determinants such as deficiencies in sanitation, poor personal hygiene and human cohabitation with domestic animals, favors the maintenance of infections, reinfections and coinfections [2,3]. These infections can result in impairments in physical and cognitive development, and eventually death [4,5], especially for young individuals.

It is estimated that one-third of the world's population is harboring intestinal parasitic infections [6,7]. In Brazil, particularly in the Northeast Region, the estimated prevalence of enteric parasites in children varies between 13% [8] and 94% [9]. In the same geographical region canine infections vary between 35% [10] and 83% [11]. In Bahia, a northeastern state, the prevalence among children from 0 to 6 years varies between 37% [12] and 77% [13], and in dogs an average of 56% has been reported [14,15]. Coinfections are common in both populations and are influenced by the natural and social environment. The complexity of these interactions of human and canine diseases is best attacked with a One Health approach.

*Giardia duodenalis* and *Ascaris lumbricoides* are the enteric parasites most frequently found in Brazilian children [13,16], while in Brazilian dogs there is a greater prevalence of *Ancylostoma* [17,18]. Income level, education, sex and age are the main variables associated with these infections [19,20].

As part of the World Health Organization neglected disease control programs [21], which include soil transmitted helminths, Brazil has implemented an Integrated Strategic Action Plan for disease elimination, including the Control of Geo-helminthiasis (ascariasis, trichuriasis and hookworm disease), effectively started in 2013 [22,23]. The program aims to reduce the enteric parasitic burden in school-age children in high risk areas. A single dose of albendazole is administered under the supervision of local health teams.

To select priority municipalities, the government considers variables such as sewage destination, availability of treated water, garbage management, HDI-M and percentage of the general population in poverty. But the criteria used in selecting schools, in several municipalities, do not serve the purposes of the program.

Regarding pets, worm control depends on the judgement of each individual veterinarian. However, many dog owners cannot afford to consult a vet or to buy over-the-counter medicine [3].

The aim of this study was to determine the prevalence of the main enteric parasites infecting children and dogs in the municipality of Ilhéus, Bahia, Brazil, and to identify the factors associated with these infections, as well as the geopolitical areas that should receive priority intervention. A better understanding of hotspots of enteric infections can contribute to the more effective selection of the places to be targeted. Such knowledge would improve efficiency and reduce costs.

## Material and Methods

### Ethical considerations

This study was approved by the Ethics Committee for the Use of Animals under protocol no 023/2015 and by the Ethics Committee for Research with Humans, under protocol CAAE 51181915.6.0000.5526, both from the State University of Santa Cruz, Bahia. This research strictly followed the Brazilian Guide for Care and Use of Animals in Teaching and Scientific Research Activities. Parents and dog owners gave written consent for fecal sample collection and administration of questionnaires.

### Study area

**Fig 1:**
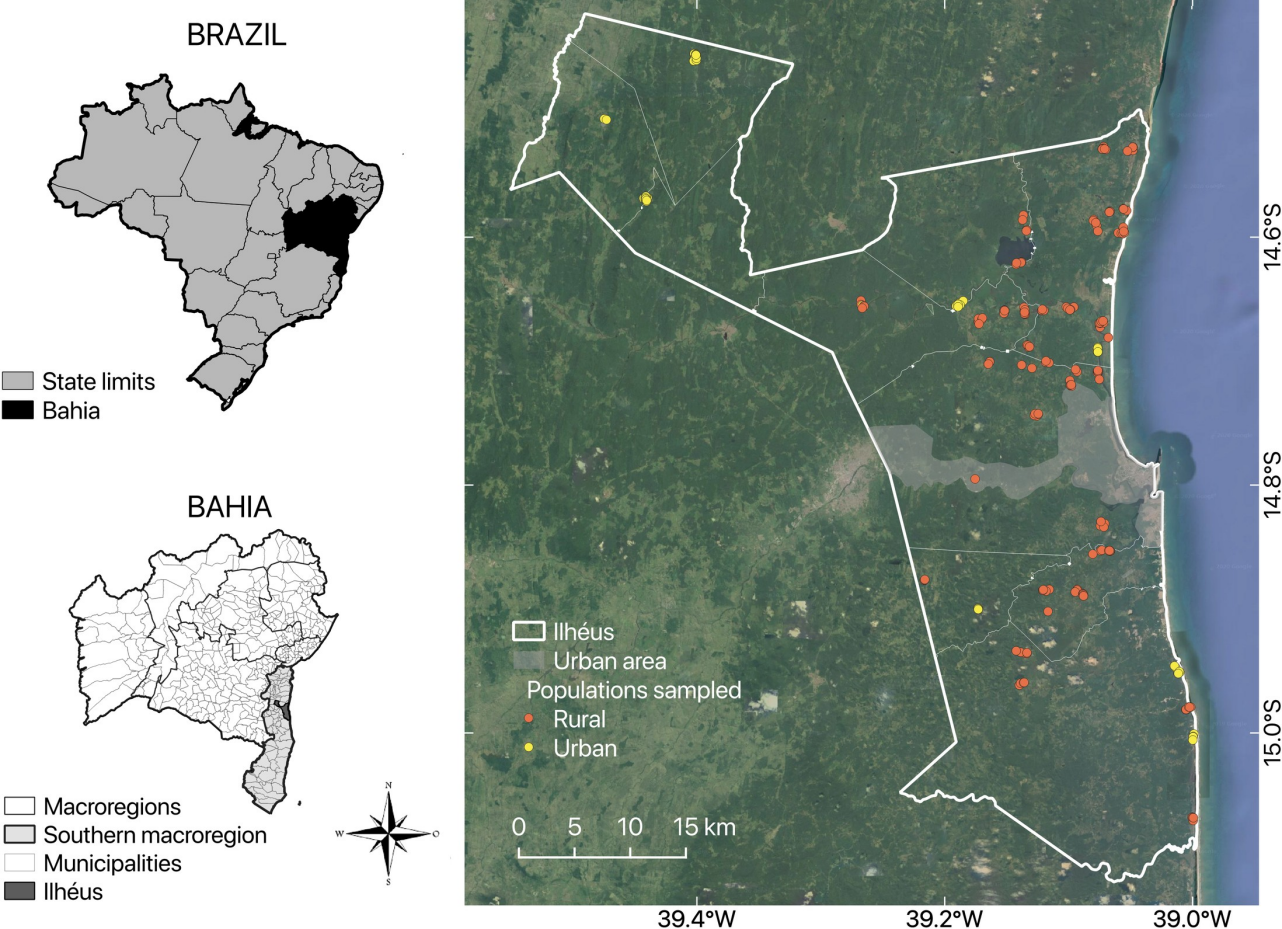
Location of Ilhéus Municipality in Bahia State, Brazil. This map was prepared using QGIS program (version 2.18).

The study was conducted in the municipality of Ilhéus (Fig 1), State of Bahia, Brazil. The municipality consists of nine rural districts and one main district, which contains the urban center and other rural areas. Each district has a main village with semi-rural characteristics.

There are 184,236 inhabitants in the municipality. A little over fifteen percent (28,955) of them are residents of rural areas, of which 72.3% receive up to half a minimum monthly salary. Only 3% of the inhabitants in the municipality has a sewage system. Most rural communities do not have access to treated water, using water from rivers, dams, artesian wells and/or natural sources for drinking and performing domestic, personal hygiene and/or leisure activities. In all communities, proper waste management is neglected by the population and the public authorities. Most streets have no pavement, and there are a large number of dogs roaming the streets. Among the schools selected through the school deworming program, only 20% are located in rural or semi-rural areas.

The State of Bahia is located in the Atlantic Rainforest Biome, with a tropical and humid climate. Annual rainfall ranges from 1000 mm to 2700 mm and mean annual compensated temperature is approximately 25°C. The city of Ilhéus has an average HDI (Human Development Index) of 0.69 and G (Gini Index) of 0.5. Its main economic activities are based on agriculture, tourism and industry [24–26].

### Study design and population

Between March and November 2016, a cross-sectional study was carried out in eight main villages and 32 rural settlements. Our target populations were children ages 1 month to 5 years and dogs. Puppies were considered to be dogs aged up to one year. Given that there is no data on the occurrence of parasitosis in this city, sample sizes were calculated based on an estimated proportion of 50% ± 5% precision with 95% confidence and a population size of 3,851 for children and 5,348 for dogs using EpiTools, https://epitools.ausvet.com.au/oneproportion). The estimated samples sizes were 350 for children and 359 for dogs. The sampling was stratified by districts and, to ensure coverage of the entire area of the villages, systematic sampling was used to select households within villages.

One fecal sample was collected from each child and each dog. It was not a precondition that the child and the dog come from the same household. In order to compare and confirm the results of the fecal samples collected by the dog owners, two fecal samples from each street per community were also collected, totaling 270 additional fecal samples. These samples were collected only to analyze the prevalence of infections and parasitic diversity.

### Samples collection

Three samples were collected per individual on alternate days using the commercial TF-Test Conventional kit (Immunoassay Industry e Comércio Ltda., Brazil) which shows high sensitivity and efficiency in the detection of enteric parasites [27,28]. The kit allows for a more practical collection and storage at room temperature, as it is recommended for collections in distant places, especially in needy populations, where the use of refrigerators is limited.

Health posts and schools, with the authorization of the respective municipal authorities, and volunteer residences received and stored the samples in a recyclable icebox. In several communities, health workers participated in the sample collection process. From the streets, samples of fresh or non-dried feces were collected and stored in sterile refrigerated stool collectors until laboratory analysis. In some communities, heavy rain resulted in fewer samples. In one rural settlement, we did not find any street samples.

### Parasitological analysis

The analyses were performed following the kit protocol (sedimentation principle) and the Faust method (1938) modified by Sloss et al. (1999) (flotation principle) [29]. The samples were divided into two aliquots, which were processed individually with each technique. Dog fecal samples collected from the streets were processed using Sheather's (1923) centrifugal-flotation technique modified by Huber et al. (2003) [30] and centrifugal-sedimentation. For all samples, slides containing one drop of Lugol's solution were analyzed in order to identify helminth eggs and protozoan cysts. To investigate *Cryptosporidium* oocysts, 20 μl of pellet were placed on slides posteriorly stained with modified Ziehl-Neelsen Technique [31]. Slides were analyzed by light microscopy with 40x and 100x objectives. *Strongyloides stercoralis* was identified through its larval stage. To be diagnosed with parasitosis, the animal must have been positive in at least one of the tests.

### Collection of epidemiological data

Interviews were conducted with parents and/or dog owners using a structured and previously tested questionnaire that included socioeconomic and behavioral questions. The assessed variables for both populations included age, sex, place of residence, education level, income level, exposure to untreated water and treatment. We also asked parents about the child’s contact with dogs, annual visit to a physician, the habit of putting hands in the mouth, washing hands after playing with soil, walking barefooted, and the source of water used to wash fruits. For the dog owners we asked questions about level of restriction, breed, and contact with other dogs. All residences were georeferenced using a Garmin GPS device.

### Statistical analysis

The prevalence and 95% confidence interval for each parasite was calculated based on the Wilson Score Interval [32] in EpiTools. Chi-square Tests were used to identify demographic, social and behavioral factors that were significantly associated with the occurrence of any parasite, Phylum-related infections and coinfections. Variables with p ≤ 0.20 in the bivariate analyses were included in the multiple logistic regression analysis. The variables in the final model were manually selected using a backward stepwise selection process. The level of significance to keep a factor in the final model was set at p≤0.05. All statistical analyses were conducted using Epi Info 7 (Centers for Disease Control and Prevention—CDC, Atlanta, USA) or the STATA statistical software release 15 (StataCorp 2017, College Station, TX). Spatialization of the overlap of infections was performed by identifying the residences and calculating the number of infections of each parasite, by district. The limits of administrative divisions were collected from Brazilian Institute of Geography and Statistics (IBGE). Thus, the maps and their graphics were done using the software QGIS (version 2.18) and Microsoft Excel.

### Feedback for communities

Reports were delivered to parents, who were instructed to present the results to their pediatricians. Dog owners were given treatment guidelines. Lectures on health education were given to the community, including adults, children, teachers and health workers; in some villages with the support of municipal schools and health posts.

## Results

### Parasitological analysis

Three hundred and nine families participated in the study. Of these, 116 households provided fecal samples from dogs, 166 provided fecal samples from children, and 27 provided samples from both. In total, 193 children and 143 dogs were sampled.

Of the 193 children surveyed, 132 (68.4%) were parasitized, predominantly with protozoa (117/132, 88.6%) in monoinfections (49/117, 41.9%) and coinfections (68/117, 58.1%). Of the 143 dogs studied, 111 (77.6%) were parasitized, predominantly with helminths (97/111, 87.4%) in monoinfections (51/97, 52.6%) and coinfections (46/97, 47.4%). Of the 270 canine fecal samples collected on the street, 199 (73.7%) were parasitized, with a predominance of helminths (174/199, 87.4%). Giardiasis, cryptosporidiosis, amoeba infections and hookworm were the most frequent infections in children and dogs, in addition to trichuriasis in dogs and ascaridiasis in children. The parasite diversity observed in both populations is shown in Table 1.

**Table 1:** Enteric parasites in feces of children and dogs in the main villages and rural settlements of the Municipality of Ilheus, BA, Brazil. March to November 2016.

	Children (N = 193)		Dogs (N = 143)		Dog fecal samples from the streets (N = 270)	
	n	% (95% CI)	n	% (95% CI)	n	% (95% CI)
**Protozoa**						
*Cryptosporidium* sp.	15	7.7 (4.4–12.5)	08	5.6 (2.4–10.7)	19	7.0 (4.2–10.8)
*Cystosospora sp*.	01	0.5 (0.0–2.8)	06	4.2 (1.5–8.9)	06	2.2 (0.8–4.8)
*Endolimax nana*	40	20.8 (15.2–27.1)	15	10.5 (5.9–16.7)	04	1.5 (0.4–3.7)
*Entamoeba coli*	47	24.3 (18.4–31.0)	13	9.1 (4.9–15.0)	27	10.0 (6.7–14.2)
*Entamoeba* complex^a^	12	6.2 (3.2–10.6)	03	2.1 (0.4–6.0)	-	-
*Giardia duodenalis*	72	37.3 (30.5–44.5)	24	16.8 (11.0–23.9)	14	5.2 (2.9–8.5)
*Iodamoeba butschlii*	08	4.1 (1.8–8.0)	-	-	-	-
*Sarcocystis sp*.	02	1.0 (0.1–3.7)	01	0.7 (0.0–3.8)	05	1.9 (0.6–4.4)
Adeleid Coccidia	-	-	-	-	02	0.7 (0.1–2.6)
*Eimeria* spp.	-	-	-	-	06	2.2 (0.8–4.8)
**Helminths**						
*Ascaris lumbricoides*	26	13.5 (8.9–19.1)	-	-	-	-
*Dipylidium caninum*	-	-	-	-	01	0.4 (0.0–2.0)
*Enterobius vermicularis*	02	1.0 (0.1–3.7)	-	-	-	-
Hookworm^b^	08	4.1 (1.8–8.0)	87	60.8 (52.3–68.9)	164	60.7 (54.6–66.6)
*Strongyloides stercoralis* (larva)	-	-	-	-	02	0.7 (0.1–2.6)
*Toxascaris leonina*	-	-	04	2.8 (0.8–7.0)	12	4.4 (2.3–7.6)
*Toxocara canis*	-	-	05	3.5 (1.1–7.9)	09	3.3 (1.5–6.2)
*Trichuris trichiura*	11	5.7 (2.9–9.9)	-	-	-	-
*Trichuris vulpis*	-	-	13	9.1 (4.9–15.0)	31	11.5 (7.9–15.9)
**Diversity of infections/host**						
One parasite	60	31.1 (24.6–38.1)	61	42.6 (34.4–51.1)	120	44.4 (38.4–50.6)
Two parasites	42	21.8 (16.2–28.2)	32	22.4 (15.8–30.1)	60	22.2 (17.4–27.7)
Three parasites	22	11.4 (7.8–16.7)	15	10.5 (6.1–16.7)	15	5.6 (3.1–9.1)
More than three parasites	8	4.1 (1.8–8.0)	3	2.1 (0.4–6.0)	4	1.5 (0.4–3.7)
Coinfections	72	37.3 (30.5–44.5)	50	35.1 (27.2–43.4)	79	29.3 (23.9–35.1)

^a^ *Entamoeba histolytica*, *E*. *dispar*, *E*. *bangladeshi* and *E*. *moshkovskii* complex.

^b^ The eggs of *Ancylostoma* spp. and *Necator americanus* were not differentiated.

### Analyses of overlapping diseases

The distribution of parasites in children and dogs by district is presented in S1 Table. The district of Olivença had the highest parasitic diversity, followed by the districts of Aritaguá and the Main District. Among all districts, the frequency of infections in the population of children varied between 50% (Inema) and 80% (Japu and Castelo Novo). In the canine population, it varied between 40% (Castelo Novo) and 100% (Inema). Non-pathogenic protozoa, *E*. *nana* and *E*. *coli* were detected in all districts.

**Fig 2:**
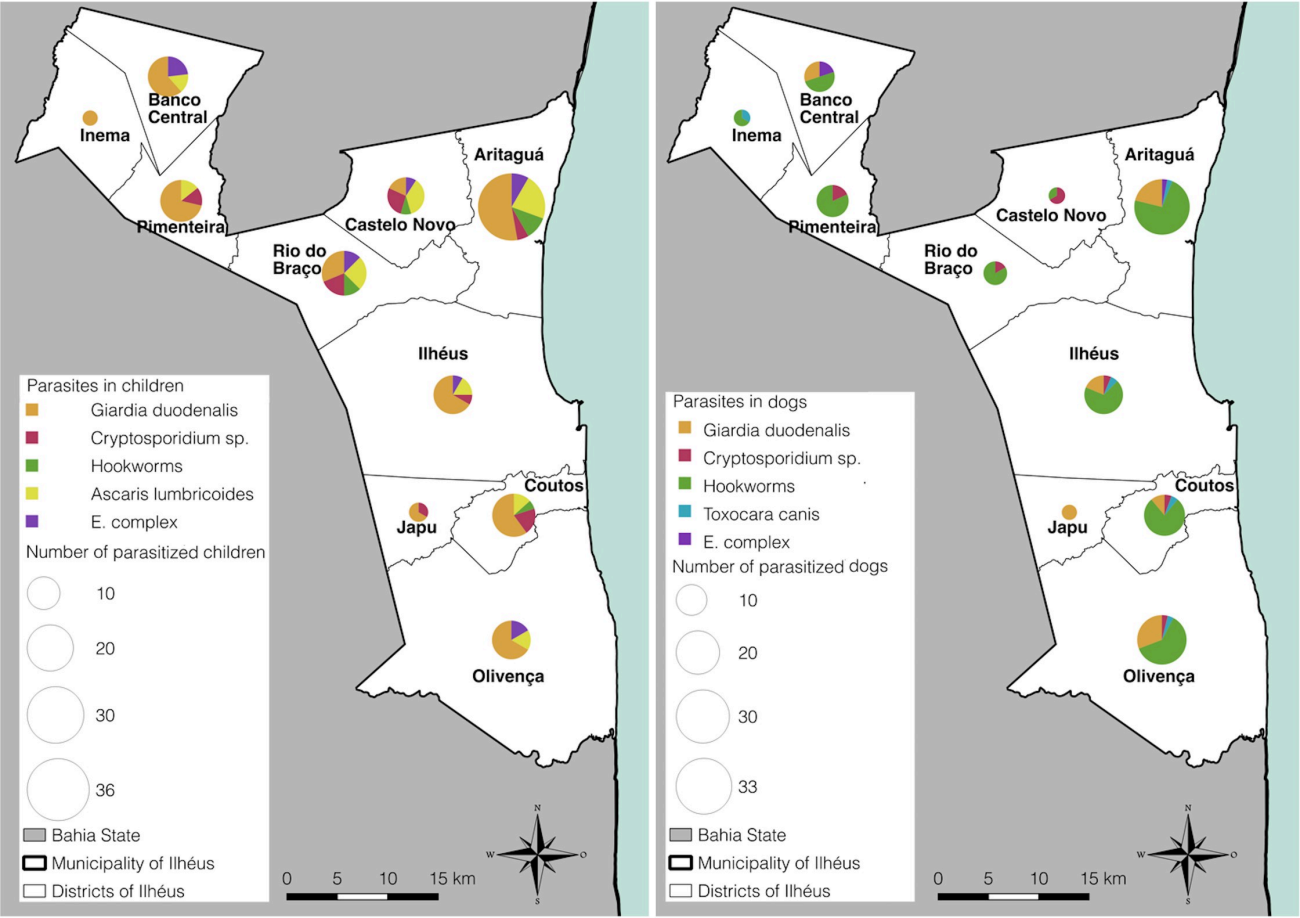
Overlapping enteric parasitic infections in the Municipality of Ilhéus at the district level. **March to November/2016.** This figure was prepared by using QGIS program (version 2.18).

Overlapping of important potential zoonotic parasites such as *Giardia*, *Cryptosporidium*, *Toxocara* and *Ancylostoma* were observed in most districts (Fig 2). A greater parasite overlap in the dog population was observed in the Aritaguá, Olivença and in the Main District, while the Districts of Aritaguá and Rio do Braço showed greater overlap of intestinal parasites in children. Thus, the districts of Aritaguá, Olivença and the Main District should be prioritized by health municipal authorities for prevention and control of intestinal parasites.

### Epidemiological analysis

**Fig 3:**
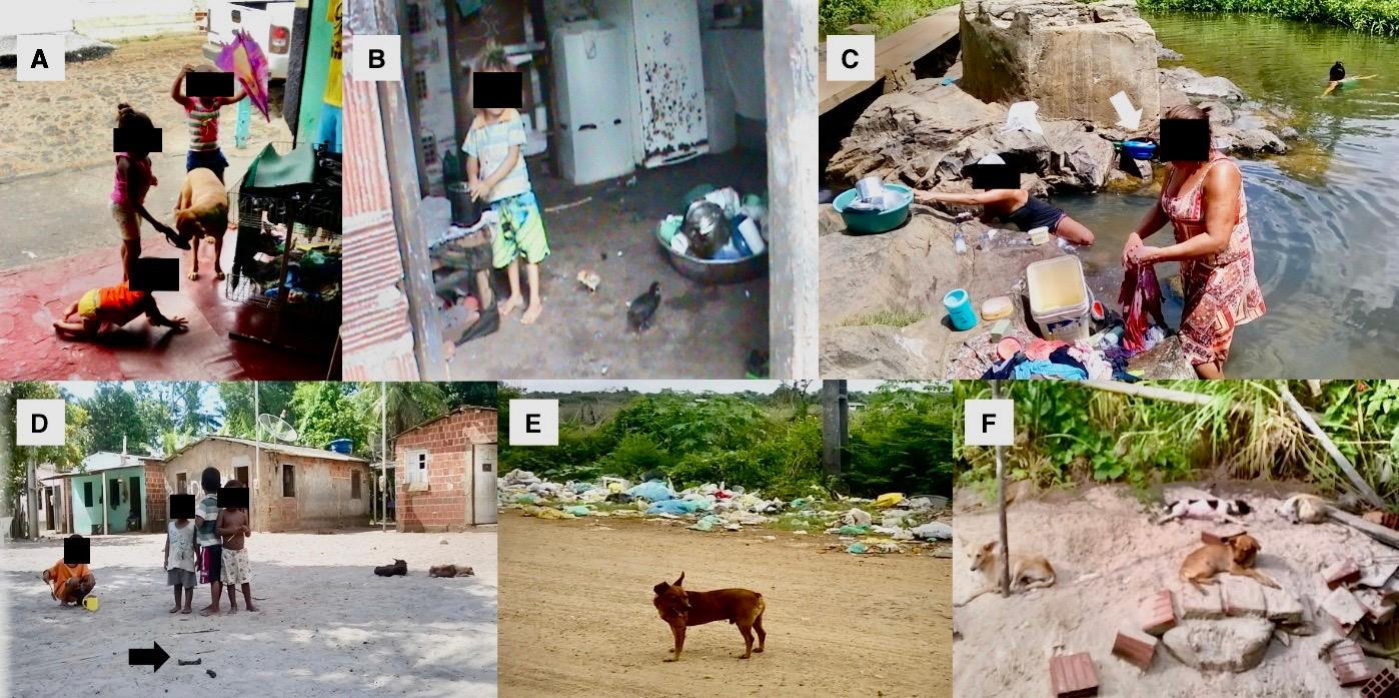
A) Interaction among children under 5 years of age with semi-restricted dog in a commercial establishment of the District of Banco Central. B) Child infected with six different species of parasites. Note the dirt floor, the storage of household utensils on the floor and the presence of domestic animals in the kitchen. District of Rio do Braço. C) Domestic activities in the Japu River. Note the presence of a basin with chicken being prepared for consumption. District of Japu. D) Children playing in a peridomiciliary environment contaminated by canine feces. District of Aritaguá. E) Semi-restricted dog in contaminated environment of the District of Couto. F) Agglomeration of unhealthy dogs in peridomicile of the Municipal Seat (Ilhéus). March to November/2016. Source: Personal File.

One hundred and five (34%) and 204 (66%) households were located in semi-rural areas (main villages) and rural areas, respectively. Regarding the level of education of the parents and dog owners, 69.8% had incomplete elementary education and 76.4% of this subset were illiterate. The majority of the population earned up to a minimum monthly salary (67.2%). The total percentage of unemployed was 50.8% and, in this group, the sources of income came from extractive and hunter-gathering activities (14.1%) or temporary jobs (85.9%). There is no sewage system in any of these communities. Most residences had independent rudimentary septic systems. Of the households that had a bathroom, 11.3% of these bathrooms drained their waste into the forest or into an open ditch and 16.2% of these bathrooms did not have a toilet. Regarding the type of water used in the households, 73.4% used untreated water from wells, springs and other groundwater sources, such as rivers, dams and/or cisterns. In several communities, the rivers were used for the accomplishment of domestic tasks, personal hygiene and swimming. In 66.5% of the households, the peridomiciliary area was not adequately sanitized, and 16.1% of dog owners did not remove feces from the peridomicile. The feces that were removed were destined for vacant lots (4.4%), garbage (25.4%), forest (46.5%) or the toilet (0.9%). Some people buried feces (13.2%) or left them in a specific place in the yard (9.6%). Groups of stray and semi-restricted dogs were observed in the streets and yards. The semi-restricted dogs often access the interior of the houses and groundwater sources. Some dogs were bathed in the bathroom and others in the rivers. These dogs had frequent interaction with the human population, especially with children, in the peridomiciliary areas and on the streets (Fig 3). We observed negligence in the disposal of garbage: there were points of garbage disposal inside and on the outskirts of the communities that were constantly accessed by dogs. Veterinary services were not accessible to the majority of the dog owners due to low purchasing power.

During the collection period, reports of abdominal pain, weight loss, diarrhea and other clinical signs related to gastrointestinal infections in children were common, especially in the community of Jairi (District of Olivença). Tables 2 and 3 show the variables included in the adjusted models (Tables 4 and 5) for both populations. The bivariate analysis tables for each outcome are presented as S2, S3, S4 and S5 Tables for the population of children and S6, S7, S8 and S9 Tables for the canine population.

**Table 2:** Bivariate analysis of factors potentially associated with intestinal infections in children from the 10 districts of the Municipality of Ilhéus, Bahia, Brazil (n = 193)^a^.

Variable		N	Infected (%)	p-value	OR	95% CI
**Enteric Parasitic Infections**						
Age	≤ 1 year	51	24 (47.1)	-	rc	-
	> 1 year	140	107 (76.4)	0.000	3.64	1.85–7.16
Level of education of the mother	HSl/Undergraduated^b^	66	41 (62.1)	-	rc	-
	E/M School^c^	117	87 (74.4)	0.08	1.76	0.92–3.38
Anual doctor consultation	Yes	63	39 (61.9)	-	rc	-
	No	127	92 (72.4)	0.14	1.61	0.85–3.07
Barefoot	No	67	41(61.2)	-	rc	-
	Yes	121	81(72.7)	0.10	1.69	0.89–3.19
Anthelminthic treatment I	Yes	129	92 (71.3)	-	rc	-
	No	57	34 (59.6)	0.12	0.59	0.31–1.14
**Helminth Infections**						
Age	≤ 1 year	51	3 (5.9)	-	rc	-
	> 1 year	140	35 (25)	0.01	5.33	1.56–18.16
Level of education of the mother	HSl/Undergraduated^b^	66	6 (9.1)	-	rc	-
	E/M School^c^	117	30 (25.6)	0.01	3.44	1.35–8.79
Anual doctor consultation	Yes	63	7 (11.1)	-	rc	-
	No	127	31 (24.4)	0.03	2.58	1.07–6.25
Barefoot	No	67	9 (13.4)	-	rc	-
	Yes	121	29 (23.4)	0.09	2.03	0.89–4.59
Hands in mouth (habit)	No	30	3 (10)	-	rc	-
	Yes	159	35 (22)	0.14	2.54	0.72–8.87
Wash hands after playing with soil	Yes	84	11 (13.1)	-	rc	-
	No	101	26 (25.7)	0.03	2.30	1.06–4.99
Exposed to untreated water	No	21	1 (4.8)	-	rc	-
	Yes	166	36 (21.7)	0.10	5.54	0.72–42.68
**Protozoa Infections**						
Age	≤ 1 year	51	23 (45.1)	-	rc	-
	> 1 year	140	93 (66.4)	0.01	2.40	1.25–4.63
Income level	> US$ 258.82^d^	16	7 (43.7)	-	rc	-
	≤ US$ 258.82	169	105 (62.1)	0.15	0.47	0.17–1.33
Contact dogs	No	37	18 (48.6)	-	rc	-
	Yes	80	51 (63.7)	0.12	1.86	0.84–4.10
Barefoot	No	67	36 (53.7)	-	rc	-
	Yes	121	78 (64.5)	0.15	1.56	0.85–2.87
Anthelminthic treatment I	Yes	129	82 (63.6)	-	rc	-
	No	57	29 (50.9)	0.11	0.59	0.31–1.11
**Enteric parasitic coinfections**						
Age	≤ 1 year	51	9 (17.6)	-	rc	-
	> 1 year	140	63 (45)	0.001	3.82	1.72–8.44
Contact dogs	No	37	11 (29.7)	-	rc	-
	Yes	80	34 (42.5)	0.19	1.74	0.76–4.01
Barefoot	No	67	18 (26.9)	-	rc	-
	Yes	121	52 (43.1)	0.03	2.05	1.07–3.93
Anthelminthic treatment I	Yes	129	52 (40.3)	-	rc	-
	No	57	17 (29.8)	0.17	0.62	0.32–1.23
Local	Semirural	73	22 (30.1)	-	rc	-
	Rural	120	50 (41.7)	0.11	1.65	0.89–3.07
Level of education of the mother	HSl/Undergraduated^b^	66	20 (30.3)	-	rc	-
	E/M School^c^	117	52 (44.4)	0.06	1.84	0.97–3.49

^a^ = Unanswered questions were discarded in the statistical analysis

^b^ = High School/Undergraduate Degree

^c^ = Elementary and Middle School

^d^ = Amount equivalent to a minimum monthly salary in Brazil, on 11/31/2016, according the Brazilian Central Bank

rc = reference category

**Table 3:** Bivariate analysis of factors potentially associated with intestinal infections in dogs from the 10 districts of the Municipality of Ilhéus, Bahia, Brazil (n = 143)^a^.

Variable		N	n (%)	p-value	OR	95% CI
**Enteric Parasitic Infections**						
Income level	> US$ 258.82^b^	35	22 (62.8)	-	rc	-
	≤ US$ 258.82	97	80 (82.4)	0.02	2.78	1.17–6.59
Antihelminthic treatment	Yes	112	83 (74.1)	-	rc	-
	No	30	27 (90)	0.08	3.14	0.88–11.14
**Helminth Infections**						
Breed	Yes	30	17 (56.7)	-	rc	-
	No	113	80 (70.8)	0.14	1.85	0.81–4.24
Income level	> US$ 258.82	35	19 (54.3)	-	rc	-
	≤ US$ 258.82	97	70 (72.2)	0.06	2.18	0.98–4.85
Antihelminthic treatment	Yes	112	71 (63.4)	-	rc	-
	No	30	25 (83.3)	0.04	2.88	1.03–8.12
**Protozoa Infections**						
Sex	Male	84	24 (28.6)	-	rc	-
	Female	59	27 (45.8)	0.04	2.18	1.05–4.17
Level of restriction	Restricted	37	9 (24.3)	-	rc	-
	Semi-restricted	106	42 (39.6)	0.10	2.04	0.87–4.75
**Enteric parasitic coinfections**						
Sex	Male	84	24 (28.6)	-	rc	-
	Female	59	26 (44.1)	0.06	1.96	0.98–4.00
Level of restriction	Restricted	37	9 (24.3)	-	rc	-
	Semi-restricted	106	41 (38.7)	0.11	1.96	0.84–4.58
Breed	Yes	30	6 (20)	-	rc	-
	No	113	44 (38.9)	0.06	2.55	0.96–6.74
Antihelminthic treatment	Yes	112	36 (32.1)	-	rc	-
	No	30	14 (46.7)	0.14	1.84	0.81–4.19

^a^ = Unanswered questions were discarded in the statistical analysis

^b^ = Amount equivalent to a minimum monthly salary in Brazil, on 11/31/2016, according the Brazilian Central Bank

rc = reference category

**Table 4:** Final multivariable models of factors associated (p≤0,05) with parasitic intestinal infections in children (n = 193)^a^ from the 10 districts of the Municipality of Ilhéus, Bahia, Brazil.

Variable		p-value	OR	95% CI
**Enteric parasitic infection**				
Age	≤ 1 year	-	rc	-
	> 1 year	0.000	3.65	1.85–7.16
**Protozoa Infection**				
Age	≤ 1 year	-	rc	-
	> 1 year	0.01	2.41	1.25–4.63
**Helminth Infection**				
Age	≤ 1 year	-	rc	-
	> 1 year	0.02	4.38	1.26–15.22
Level of education of the mother	HS/Undergraduated^b^	-	rc	-
	E/M School^c^	0.02	3.03	1.17–7.84
**Enteric parasitic coinfections**				
Age	≤ 1 year	-	rc	-
	> 1 year	0.001	3.82	1.73–8.44

^a^ = Unanswered questions were discarded in the statistical analysis

^b^ = High School/Undergraduate Degree

^c^ = Elementary and Middle School

rc = reference category

**Table 5:** Final multivariable models of factors associated (p≤0,05) with parasitic intestinal infections in dogs (N = 143)^a^ from the 10 districts of the Municipality of Ilhéus, Bahia, Brazil.

Variable		p-value	OR	95% CI
**Enteric parasitic infection**				
Income level	> US$ 258.82^b^	-	rc	-
	≤ US$ 258.82	0.02	2.78	1.17–6.59
**Protozoa Infection**				
Sex	Male	-	rc	-
	Female	0.04	2.18	1.05–4.17
**Helminth Infection**				
Antihelminthic treatment	Yes	-	rc	-
	No	0.05	2.89	1.03–8.12
**Enteric parasitic coinfections**				
Sex	Male	-	rc	-
	Female	0.04	2.18	1.03–4.35
Breed	Yes	-	rc	-
	No	0.04	2.76	1.03–7.41

^a^ = Unanswered questions were discarded in the statistical analysis

^b^ = Amount equivalent to a minimum monthly salary in Brazil, on 11/31/2016, according the Brazilian Central Bank

rc = reference category

## Discussion

The high parasitic frequency and diversity, including important zoonosis, observed in coastal districts, which house the majority of the human population, indicate a need for urgent sanitary interventions to reduce the prevalence and risk of disease transmission. Mass treatment, as advocated by the national soil-transmitted disease control program, has up to now more concentrated in urban areas, but the need is also urgent in rural areas [22,23].

The cultural impact on the spread of parasites is striking: poverty, which leads to inadequate personal and environmental hygiene, compounded by negligence of the canine population, all perpetuate themselves through generations. Unsanitary conditions and poor personal hygiene have become the norm, indicating that not only changes in the health infrastructure, but also changes in human behavior are necessary [33,34].

In the case of Ilhéus, as in other communities in tropical areas, many factors contribute to the maintenance of parasites in the environment, such as: a) climatic conditions favoring the survival of parasites [3,35]; b) the interaction between cycles of transmission, as well as the frequent contact between dogs and humans, which can favor the dissemination of zoonoses [1,2]; c) the contact of synanthropic animals, insects and dogs with the trash dumps; d) the lack of adequate garbage disposal services; e) the large number of common grounds favoring the interaction of infected and non-infected individuals and animals; f) the large number of semi-restricted dogs [36,37]; g) and the precarious hygienic-sanitary conditions of these communities, which was confirmed by the high occurrence of non-pathogenic protozoa [12,34]. In this context, the implementation of preventive actions must be a forceful and continuing process.

In the last 20 years, several studies have emphasized that protozoan infections are a major challenge for Latin American children, including Brazilian children [12,13,16,38]. Our study confirms and expands upon these earlier efforts. It follows that actions targeting protozoa infections must be developed and prioritized over helminthiasis. The endemic giardiasis, as well as the high prevalence of non-pathogenic amoebae, which are an indicator of inadequate sanitation, demand investments in the sanitary structure and urgent access to potable water. But, the causes of giardiasis and cryptosporidiosis in children and dogs probably go beyond consumption and exposure to untreated water. The constant exposure to different potential transmission cycles, such as contact with infected individuals (asymptomatic or not), pets, farm animals and wild animals, and contact with contaminated environments inside and outside the households, all require massive action in health education [1,2,39–43].

The low prevalence of helminths is a reflection of the partial success of the Brazilian public program of prevention and control [31,32]. Mothers in rural areas are learning that giving medicine to their children every six months can give the children a healthier life.

In the rural and semirural areas of Ilhéus, we can affirm that mass antihelminthic treatment is not the driving force of improvement; the driving force is information—awareness at the family level of the need for periodic medication. But, despite the impact of information, we have to consider that helminthiasis is still present. Helminthiasis leads to developmental disorders in children. The active agent here is constant reinfection—according to the testimony of many mothers.

The current program targets schoolchildren between 5 and 14 [22,23]. Within that population, less than 20% are attending rural schools. We believe that children in both urban and rural areas would benefit if the program included children under five, since children of all ages up to 14 live in the same families.

Turning our attention now to enteric parasitic infections in dogs, poor Brazilian populations usually neglect the treatment of infections in their dogs, particularly because of the low purchasing power and low awareness of the risk of zoonosis transmission [3,44–46]. We observed that low income impacts the cultural aspect of animal health management. Garlic and mastruz tea, used to treat human worms, were also used for the same purpose in dogs. In addition, the telltale signs of infection in the feces of infected dogs are not always observed by owners. Consequently, the dogs go untreated. In these communities, health workers are making progress towards better management of the canine population. They are teaching the owners that deworming is necessary for the health of the dogs, and in fact to the health of humans in the same household, because an infected dog produces contaminated feces. However, deworming is performed once a year or only when the animals are diseased, which is not enough when parasites are present in the environment. To control parasites in dogs, the contamination of the streets and yards must also be controlled.

As for the risk factors for enteric parasites, children older than one year are at higher risk of infection. By that age children become exposed to more sources of environmental contamination [16,20,38]. There are also social determinants in the transmission of disease. These determinants were pointed out by the bivariate analysis and must be considered in the design of preventive strategies that focus, especially, on personal hygiene habits and treatment of infections. This need is also implicit in the high infection rate in children under one year of age, which requires approaches with the adults who care for them. Among other sources of infection are contaminated water, food and the environment. [12].

The level of maternal education is a significant factor in parasitic intestinal infections, according to other studies [38,47,48] and proved to be relevant in this study. Its greater relevance to helminth infections in children points to two possible explanations: a) in many villages mothers were aware of basic and adequate hygiene habits, but still neglected to comply with the information received or pass it on to their children, which can be exemplified by the large number of children usually barefoot, or who put their hands in their mouths, or who do not wash them after playing in the soil; and b) the administration of antihelminthics to children only once a year or only when the mother suspects that a child is infected.

The association of female dogs with enteric parasite coinfections can be related to the longer periods of immunosuppression that females go through, caused by multiple gestations and puppies feeding, added to isolation during estrus, and poor diet, compared to males. These conditions can favor autoinfection by hookworm and ascarid larvae, consequently increasing environmental contamination of yards and streets. The chaining of the dogs in contaminated yards where children play can also lead to exposure of the dogs to infection by protozoa [49–51].

When the municipality prepares to reapply mass antihelminthic treatment, identification of areas of highest occurrence and greatest infection overlap, especially of the most relevant zoonotic infections, can guide logistics and optimize the performance and results of the current program. Prioritizing the most affected areas, as well as the highest risk areas may result in a faster and more significant reduction in the prevalence of these diseases in humans and animals.

Preventive programs must mandatorily and simultaneously include interventions on human and animal populations and in the environment. We further suggest: a) comprehensive sterilization of domestic dogs in rural areas as a public policy strategy, which can result in decrease of the number of puppies, and consequently street animals and contaminated feces in the environment; b) distribution of antihelminthics to the canine population during rabies vaccination campaigns; c) guidance to tutors on the use of antiheminthics; d) a partnership between Municipality services, universities and local laboratories to develop strategies for the management of the canine population and surveillance of zoonoses; e) integration of professionals from different areas in the delineation and execution of preventive actions based on the principle of One Health; f) effective community participation. All of these measures, added to the actions previously proposed, must take into account cultural factors and social determinants, which are often neglected in the planning of the current interventions.

Changing the cultural habits and lifestyles of human population will not be an easy demand. It should also be considered that the economic situation of Brazil and other tropical countries, generally, allows for little hope for real short-term infrastructure improvements. In this case, it is clear that continuous health education programs may be more successful and cost-effective, compared to government anti-poverty programs such as Bolsa Familia, as a way to reduce prevalence of transmissible diseases in rural affected communities.

Studies that demonstrate social inequality in health are not new [52]. The strong association of poverty with the endemic of enteric parasites is very clear in this study. This study calls into question the effectiveness of the anti-poverty program Bolsa Família [53] on the poverty and health of these populations and on which the majority of the rural population of Ilhéus depends to survive. Since 2003, this program has sought to raise the health condition of populations in a state of poverty and extreme poverty through the conditional cash transfer to families that have a monthly per capita incomes lower than U$ 45.29 [54,55] In fact, the Bolsa Família has only been a palliative solution for the complexity of the problems related to poverty. Government statistics that show a reduction of poverty through these payments are misleading as such payments have not in fact changed the conditions under which these families live. We realize that the improvement of quality of life and, consequently, of health depends on the convergence between affirmative income actions and actions focused on improving the sanitary conditions to which these populations are subjected. The precarious health of these environments imposes the constant exposure of these individuals to agents that cause infectious and parasitic diseases. In this context, these actions could then work as more effective preventive measures to combat these diseases.

Our study was limited by a smaller sample size than anticipated due to the lack of interest in certain participants, which limited our ability to find statistically significant associations in our multivariate analysis.

In conclusion, the coastal districts of Ilhéus should be prioritized for the implementation of the national program for the prevention and control of soil-transmitted diseases, as they have higher prevalence and diversity of enteric parasites in human and canine populations. The actors associated with infections must be considered in the design of health interventions. The adoption of a holistic approach, following the principle of One Health, to combat these parasites is fundamental to repair the health deficiencies that lead to the perpetuation of these infections. Preventive programs must mandatorily control canine population and promote health education, focusing particularly on habits of hygiene and on the proper sanitation of the yards. Analysis of water sources, including seasonal studies, are necessary to better understand the epidemiology of these parasites.

## Supporting information

S1 Table Presence of enteric parasites in dogs and children from all the districts of Ilhéus, Bahia, Brazil. **March to November/2016.** C = children; D = dogs; AR = Aritaguá; BC = Banco Central; CN = Castelo Novo; CO = Couto; IN = Inema; JA = Japu; OL = Olivença; PI = Pimenteira; RB = Rio do Braço; SE = Main district. Countain the urban center.The findings of all fecal samples from dogs were included in this table.
(PDF)

S2 Table Univariate analysis of factors potentially associated with enteric parasitic infections in children from the 10 districts of the Municipality of Ilhéus, Bahia, Brazil. (n = 193)*. * = Unanswered questions were discarded in the statistical analysis ** = High School/Undergraduate Degree *** = Elementary and Middle School **** = Amount equivalent to a minimum monthly salary in Brazil, on 11/31/2016, according the Brazilian Central Bank rc = reference category.
(PDF)

S3 Table Univariate analysis of factors potentially associated with helminth infections in children from the 10 districts of the Municipality of Ilhéus, Bahia, Brazil. (n = 193)*. * = Unanswered questions were discarded in the statistical analysis ** = High School/Undergraduate Degree *** = Elementary and Middle School **** = Amount equivalent to a minimum monthly salary in Brazil, on 11/31/2016, according the Brazilian Central Bank rc = reference category.
(PDF)

S4 Table Univariate analysis of factors potentially associated with protozoa infections in children from the 10 districts of the Municipality of Ilhéus, Bahia, Brazil. (n = 193)* * = Unanswered questions were discarded in the statistical analysis ** = High School/Undergraduate Degree *** = Elementary and Middle School **** = Amount equivalent to a minimum monthly salary in Brazil, on 11/31/2016, according the Brazilian Central Bank rc = reference category.
(PDF)

S5 Table Univariate analysis of factors potentially associated with enteric parasitic coinfections in children from the 10 districts of the Municipality of Ilhéus, Bahia, Brazil. (n = 193)* * = Unanswered questions were discarded in the statistical analysis ** = High School/Undergraduate Degree *** = Elementary and Middle School **** = Amount equivalent to a minimum monthly salary in Brazil, on 11/31/2016, according the Brazilian Central Bank rc = reference category.
(PDF)

S6 Table Univariate analysis of factors potentially associated with enteric parasite infection in dogs from the 10 districts of the Municipality of Ilhéus, Bahia, Brazil (n = 143)*. * = Unanswered questions were discarded in the statistical analysis ** = High School/Undergraduate Degree *** = Elementary and Middle School **** = Amount equivalent to a minimum monthly salary in Brazil, on 11/31/2016, according the Brazilian Central Bank rc = reference category.
(PDF)

S7 Table Univariate analysis of factors potentially associated with helminth infections in dogs from the 10 districts of the Municipality of Ilhéus, Bahia, Brazil (n = 143)*. * = Unanswered questions were discarded in the statistical analysis ** = High School/Undergraduate Degree *** = Elementary and Middle School **** = Amount equivalent to a minimum monthly salary in Brazil, on 11/31/2016, according the Brazilian Central Bank rc = reference category.
(PDF)

S8 Table Univariate analysis of factors potentially associated with protozoa infections in dogs from the 10 districts of the Municipality of Ilhéus, Bahia, Brazil (n = 143)*. * = Unanswered questions were discarded in the statistical analysis ** = High School/Undergraduate Degree *** = Elementary and Middle School **** = Amount equivalent to a minimum monthly salary in Brazil, on 11/31/2016, according the Brazilian Central Bank rc = reference category.
(PDF)

S9 Table Bivariate analysis of factors potentially associated with coinfections in dogs from the 10 districts of the Municipality of Ilhéus, Bahia, Brazil (n = 143)*. * = Unanswered questions were discarded in the statistical analysis ** = High School/Undergraduate Degree *** = Elementary and Middle School **** = Amount equivalent to a minimum monthly salary in Brazil, on 11/31/2016, according the Brazilian Central Bank rc = reference category.
(PDF)
