# Supplementary material for: Enteric parasitic infections in children and dogs in resource-poor communities in northeastern Brazil: Identifying priority prevention and control areas
Source: PLoS Negl Trop Dis. 2020 Jun 9;14(6):e0008378. doi: 10.1371/journal.pntd.0008378 (PMC7282628; doi:10.1371/journal.pntd.0008378)
Supplement: S1 Table — March to November/2016. C = children; D = dogs; AR = Aritaguá; BC = Banco Central; CN = Castelo Novo; CO = Couto; IN = Inema; JA = Japu; OL = Olivença; PI = Pimenteira; RB = Rio do Braço; SE = Main district. Countain the urban center.The findings of all fecal samples from dogs were included in this table. (PDF) [file pntd.0008378.s001.pdf]

S1 – Table

S1 Table – Presence of enteric parasites in dogs and children from all the districts of Ilhéus, Bahia, Brazil. March to November/2016

Enteric Parasites	Districts									
	**OL**	**SE**	**AR**	**PI**	**CO**	**RB**	**BC**	**CN**	**JA**	**IN**
Giardia duodenalis	C, D	C, D	C, D	C	C, D	C, D	C, D	C, D	C, D	C, D
Entamoeba coli	C, D	C, D	C, D	C, D	C, D	C	C, D	C, D	C	C, D
Endolimax nana	C, D	C, D	C, D	C	C	C	C, D	C, D	C	C
Cryptosporidium spp.	D	C, D	C, D	C, D	C, D	C, D	-	C, D	C	-
Cystosospora spp.	D	D	C, D	D	-	D	D	-	-	-
E complex*	C	C	C, D	-	C	C	C, D	-	-	-
Iodamoeba butschlii	C	C	C	C	C	-	C	-	-	-
Sarcocystis spp.	-	D	-	-	-	-	-	-	-	-
Hookworm**	D	D	C, D	D	C, D	C, D	D	C, D	D	D
Trichuris spp.	D	D	C, D	C, D	D	C	D	D	D	-
Ascaris lumbricoides	C	C	C	C	C	C	C	C	-	-
Toxocara canis	D	D	D	-	D	-	D	D	D	-
Toxascaris leonina	D	D	D	D	D	D	-	-	-	-
Enterobius vermicularis	-	-	-	C	-	C	-	-	-	-
Dipylidium caninum	D	-	-	-	-	-	-	-	-	-
Strongyloides stercoralis	-	-	-	D	-	-	-	-	-	-

C = children; D = dogs; AR = Aritaguá; BC = Banco Central; CN = Castelo Novo; CO = Couto; IN = Inema; JA = Japu; OL = Olivença; PI = Pimenteira; RB = Rio do Braço; SE = Main district. Countain the urban center. The findings of all fecal samples from dogs were included in this table.
