# Supplementary material for: Enteric parasitic infections in children and dogs in resource-poor communities in northeastern Brazil: Identifying priority prevention and control areas
Source: PLoS Negl Trop Dis. 2020 Jun 9;14(6):e0008378. doi: 10.1371/journal.pntd.0008378 (PMC7282628; doi:10.1371/journal.pntd.0008378)
Supplement: S2 Table — (n = 193)*. * = Unanswered questions were discarded in the statistical analysis ** = High School/Undergraduate Degree *** = Elementary and Middle School **** = Amount equivalent to a minimum monthly salary in Brazil, on 11/31/2016, according the Brazilian Central Bank rc = reference category. (PDF) [file pntd.0008378.s002.pdf]

S2_Table

S2 Table – Univariate analysis of factors potentially associated with enteric parasitic infections in children from the 10 districts of the Municipality of Ilhéus, Bahia, Brazil. (n=193)*

Variable		n	Infected (%)	p-value	OR	95% CI
Age	≤ 1 year	51	24 (47.1)	-	rc	-
	> 1 year	140	107 (76.4)	0.000	3.64	1.85-7.16
Sex	Female	86	58 (67.4)	-	rc	-
	Male	105	73 (69.5)	0.76	1.10	0.59-2.03
Local	Semirural	73	47 (64.4)	-	rc	-
	Rural	120	85 (70.8)	0.35	1.34	0.72-2.49
Level of education of the mother	HSI/Undergraduated**	66	41 (62.1)	-	rc	-
	E/M School***	117	87 (74.4)	0.08	1.76	0.92-3.38
Income level	> US$ 258.82****	16	9 (56.2)	-	rc	-
	≤ US$ 258.82	169	118 (69.8)	0.27	0.55	0.19-1.57
Contact dogs	No	37	23 (62.2)	-	rc	-
	Yes	80	59 (73.7)	0.21	1.71	0.74-3.92
Exposed to untreated water	No	21	12 (57.1)	-	rc	-
	Yes	166	116 (69.9)	0.24	1.74	0.68-4.39
Anual doctor consultation	Yes	63	39 (61.9)	-	rc	-
	No	127	92 (72.4)	0.14	1.61	0.85-3.07
Barefoot	No	67	41(61.2)	-	rc	-
	Yes	121	81(72.7)	0.10	1.69	0.89-3.19
Hands in mouth (habit)	No	30	20 (66.7)	-	rc	-
	Yes	159	109 (68.5)	0.83	1.09	0.47-2.50
Wash hands after playing with soil	Yes	84	56 (66.7)	-	rc	-
	No	101	70 (69.3)	0.70	1.12	0.61-2.10
Type of water used to wash fruits	Treated	31	23 (69.7)	-	rc	-
	Untreated	146	102 (68.9)	0.93	0.96	0.42-2.19
Anthelminthic treatment □	Yes	129	92 (71.3)	-	rc	-
	No	57	34 (59.6)	0.12	0.59	0.31-1.14

*= Unanswered questions were discarded in the statistical analysis
**= High School/Undergraduate Degree
***= Elementary and Middle School
****= Amount equivalent to a minimum monthly salary in Brazil, on 11/31/2016, according the Brazilian Central Bank
rc = reference category
