# Supplementary material for: Enteric parasitic infections in children and dogs in resource-poor communities in northeastern Brazil: Identifying priority prevention and control areas
Source: PLoS Negl Trop Dis. 2020 Jun 9;14(6):e0008378. doi: 10.1371/journal.pntd.0008378 (PMC7282628; doi:10.1371/journal.pntd.0008378)
Supplement: S3 Table — (n = 193)*. * = Unanswered questions were discarded in the statistical analysis ** = High School/Undergraduate Degree *** = Elementary and Middle School **** = Amount equivalent to a minimum monthly salary in Brazil, on 11/31/2016, according the Brazilian Central Bank rc = reference category. (PDF) [file pntd.0008378.s003.pdf]

S3_Table

S3 Table – Univariate analysis of factors potentially associated with helminth infections in children from the 10 districts of the Municipality of Ilhéus, Bahia, Brazil. (n=193)*

Variable		n	Infected (%)	p-value	OR	95% CI
Age	≤ 1 year	51	3 (5.9)	-	rc	-
	> 1 year	140	35 (25)	0.01	5.33	1.56-18.16
Sex	Female	86	16 (18.6)	-	rc	-
	Male	105	22 (20.9)	0.69	1.16	0.56-2.38
Local	Semirural	73	14 (19.2)	-	rc	-
	Rural	120	24 (20)	0.89	1.05	0.50-2.19
Level of education of the mother	HSl/Undergraduated**	66	6 (9.1)	-	rc	-
	E/M School***	117	30 (25.6)	0.01	3.44	1.35-8.79
Income level	> US$ 258.82****	16	2 (12.5)	-	rc	-
	≤ US$ 258.82	169	35 (20.7)	0.44	0.55	0.12-2.52
Contact dogs	No	37	7 (18.9)	-	rc	-
	Yes	80	19 (23.7)	0.56	1.33	0.50-3.52
Exposed to untreated water	No	21	1 (4.8)	-	rc	-
	Yes	166	36 (21.7)	0.10	5.54	0.72-42.68
Anual doctor consultation	Yes	63	7 (11.1)	-	rc	-
	No	127	31 (24.4)	0.03	2.58	1.07-6.25
Barefoot	No	67	9 (13.4)	-	rc	-
	Yes	121	29 (23.4)	0.09	2.03	0.89-4.59
Hands in mouth (habit)	No	30	3 (10)	-	rc	-
	Yes	159	35 (22)	0.14	2.54	0.72-8.87
Wash hands after playing with soil	Yes	84	11 (13.1)	-	rc	-
	No	101	26 (25.7)	0.03	2.30	1.06-4.99
Type of water used to wash fruits	Treated	31	6 (18.2)	-	rc	-
	Untreated	146	30 (20.3)	0.79	1.14	0.43-3.02
Anthelminthic treatment	Yes	129	27 (20.9)	-	rc	-
	No	57	11 (19.3)	0.80	0.90	0.41-1.97

*= Unanswered questions were discarded in the statistical analysis
**= High School/Undergraduate Degree
***= Elementary and Middle School
****= Amount equivalent to a minimum monthly salary in Brazil, on 11/31/2016, according the Brazilian Central Bank
rc = reference category
