# Supplementary material for: Enteric parasitic infections in children and dogs in resource-poor communities in northeastern Brazil: Identifying priority prevention and control areas
Source: PLoS Negl Trop Dis. 2020 Jun 9;14(6):e0008378. doi: 10.1371/journal.pntd.0008378 (PMC7282628; doi:10.1371/journal.pntd.0008378)
Supplement: S4 Table — (n = 193)* * = Unanswered questions were discarded in the statistical analysis ** = High School/Undergraduate Degree *** = Elementary and Middle School **** = Amount equivalent to a minimum monthly salary in Brazil, on 11/31/2016, according the Brazilian Central Bank rc = reference category. (PDF) [file pntd.0008378.s004.pdf]

S4_Table

S4 Table – Univariate analysis of factors potentially associated with protozoa infections in children from the 10 districts of the Municipality of Ilhéus, Bahia, Brazil. (n=193)*

Variable		n	Infected (%)	p-value	OR	95% CI
Age	≤ 1 year	51	23 (45.1)	-	rc	-
	> 1 year	140	93 (66.4)	0.01	2.40	1.25-4.63
Sex	Female	86	51 (59.3)	-	rc	-
	Male	105	65 (61.9)	0.71	1.11	0.62-1.99
Local	Semirural	73	44 (60.3)	-	rc	-
	Rural	120	73 (60.8)	0.94	1.02	0.56-1.85
Level of education of the mother	HSI/Undergraduated**	66	41 (62.1)	-	rc	-
	E/M School***	117	74 (63.2)	0.88	1.05	0.56-1.95
Income level	> US$ 258.82****	16	7 (43.7)	-	rc	-
	≤ US$ 258.82	169	105 (62.1)	0.15	0.47	0.17-1.33
Contact dogs	No	37	18 (48.6)	-	rc	-
	Yes	80	51 (63.7)	0.12	1.86	0.84-4.10
Exposed to untreated water	No	21	12 (57.1)	-	rc	-
	Yes	166	101 (60.8)	0.74	1.16	0.46-2.92
Anual doctor consultation	Yes	63	38 (60.3)	-	rc	-
	No	127	78 (61.4)	0.88	1.05	0.56-1.94
Barefoot	No	67	36 (53.7)	-	rc	-
	Yes	121	78 (64.5)	0.15	1.56	0.85-2.87
Hands in mouth (habit)	No	30	18 (60)	-	rc	-
	Yes	159	96 (60.4)	0.28	1.5	0.72-3.11
Wash hands after playing with soil	Yes	84	53 (63.1)	-	rc	-
	No	101	59 (58.4)	0.52	0.82	0.45-1.49
Type of water used to wash fruits	Treated	31	21 (63.4)	-	rc	-
	Untreated	146	89 (60.1)	0.71	0.86	0.39-1.88
Anthelminthic treatment [illegible]	Yes	129	82 (63.6)	-	rc	-
	No	57	29 (50.9)	0.11	0.59	0.31-1.11

*= Unanswered questions were discarded in the statistical analysis
**= High School/Undergraduate Degree
***= Elementary and Middle School
****= Amount equivalent to a minimum monthly salary in Brazil, on 11/31/2016, according the Brazilian Central Bank
rc = reference category
