# Supplementary material for: Enteric parasitic infections in children and dogs in resource-poor communities in northeastern Brazil: Identifying priority prevention and control areas
Source: PLoS Negl Trop Dis. 2020 Jun 9;14(6):e0008378. doi: 10.1371/journal.pntd.0008378 (PMC7282628; doi:10.1371/journal.pntd.0008378)
Supplement: S5 Table — (n = 193)* * = Unanswered questions were discarded in the statistical analysis ** = High School/Undergraduate Degree *** = Elementary and Middle School **** = Amount equivalent to a minimum monthly salary in Brazil, on 11/31/2016, according the Brazilian Central Bank rc = reference category. (PDF) [file pntd.0008378.s005.pdf]

S5_Table

S5 Table – Univariate analysis of factors potentially associated with enteric parasitic coinfections in children from the 10 districts of the Municipality of Ilhéus, Bahia, Brazil. (n=193)*

Variable		n	Infected (%)	p-value	OR	95% CI
Age	≤ 1 year	51	9 (17.6)	-	rc	-
	> 1 year	140	63 (45)	0.001	3.82	1.72-8.44
Sex	Female	86	29 (33.7)	-	rc	-
	Male	105	43 (40.9)	0.31	1.36	0.75-2.46
Local	Semirural	73	22 (30.1)	-	rc	-
	Rural	120	50 (41.7)	0.11	1.65	0.89-3.07
Level of education of the mother	HSI/Undergraduated**	66	20 (30.3)	-	rc	-
	E/M School***	117	52 (44.4)	0.06	1.84	0.97-3.49
Income level	> US$ 258.82****	16	4 (25)	-	rc	-
	≤ US$ 258.82	169	66(39)	0.27	0.52	0.16-1.68
Contact dogs	No	37	11 (29.7)	-	rc	-
	Yes	80	34 (42.5)	0.19	1.74	0.76-4.01
Exposed to untreated water	No	21	10 (47.6)	-	rc	-
	Yes	166	60 (36.1)	0.31	0.62	0.23-1.55
Anual doctor consultation	Yes	63	23 (36.5)	-	rc	-
	No	127	49 (38.6)	0.78	1.09	0.58-2.04
Barefoot	No	67	18 (26.9)	-	rc	-
	Yes	121	52 (43.1)	0.03	2.05	1.07-3.93
Hands in mouth (habit)	No	30	10 (33.3)	-	rc	-
	Yes	159	60 (37.7)	0.64	1.21	0.53-2.76
Wash hands after playing with soil	Yes	84	27 (32.1)	-	rc	-
	No	101	41 (40.6)	0.24	1.44	0.79-2.64
Type of water used to wash fruits	Treated	31	14 (42.4)	-	rc	-
	Untreated	146	53 (35.8)	0.48	0.75	0.35-1.63
Anthelminthic treatment ▯	Yes	129	52 (40.3)	-	rc	-
	No	57	17 (29.8)	0.17	0.62	0.32-1.23

*= Unanswered questions were discarded in the statistical analysis
**= High School/Undergraduate Degree
***= Elementary and Middle School
****= Amount equivalent to a minimum monthly salary in Brazil, on 11/31/2016, according the Brazilian Central Bank
rc = reference category
