# Supplementary material for: Enteric parasitic infections in children and dogs in resource-poor communities in northeastern Brazil: Identifying priority prevention and control areas
Source: PLoS Negl Trop Dis. 2020 Jun 9;14(6):e0008378. doi: 10.1371/journal.pntd.0008378 (PMC7282628; doi:10.1371/journal.pntd.0008378)
Supplement: S6 Table — * = Unanswered questions were discarded in the statistical analysis ** = High School/Undergraduate Degree *** = Elementary and Middle School **** = Amount equivalent to a minimum monthly salary in Brazil, on 11/31/2016, according the Brazilian Central Bank rc = reference category. (PDF) [file pntd.0008378.s006.pdf]

S6_Table

S6 Table – Univariate analysis of factors potentially associated with enteric parasite infection in dogs from the 10 districts of the Municipality of Ilhéus, Bahia, Brazil (n=143)*.

Variable		n	Infected (%)	p-value	OR	95% CI
Age	< 1 year	53	42 (79.2)	-	rc	-
	> 1 year	90	69 (76.7)	0.72	0.86	0.37-1.96
Sex	Female	59	48 (81.3)	-	rc	-
	Male	84	63 (75)	0.37	0.69	0.30-1.56
Level of restriction	Restricted	37	28 (75.7)	-	rc	-
	Semirestricted	106	83 (78.3)	0.74	1.15	0.48-2.80
Breed	Yes	30	21 (70)	-	rc	-
	No	113	90 (79.6)	0.26	1.68	0.68-4.14
Local	Semirural	41	29 (70.7)	-	rc	-
	Rural	102	82 (80.4)	0.21	1.69	0.73-3.89
Level of education of dog owners	HS/Undergraduated**	30	24 (80)	-	rc	-
	E/M School***	98	76 (77.5)	0.77	0.86	0.31-2.37
Income level	> US$ 258.82****	35	22 (62.8)	-	rc	-
	≤ US$ 258.82	97	80 (82.4)	0.02	2.78	1.17-6.59
Contact with other dogs	No	30	22 (73.3)	-	rc	-
	Yes	113	89 (78.8)	0.53	1.34	0.53-3.40
Exposure to untreated water	No	18	12 (66.7)	-	rc	-
	Yes	122	96 (78.7)	0.26	1.84	0.63-5.39
Anthelminthic treatment	Yes	112	83 (74.1)	-	rc	-
	No	30	27 (90)	0.08	3.14	0.88-11.14

*= Unanswered questions were discarded in the statistical analysis
**= High School/Undergraduate Degree
***= Elementary and Middle School
****= Amount equivalent to a minimum monthly salary in Brazil, on 11/31/2016, according the Brazilian Central Bank
rc = reference category
