# Supplementary material for: Enteric parasitic infections in children and dogs in resource-poor communities in northeastern Brazil: Identifying priority prevention and control areas
Source: PLoS Negl Trop Dis. 2020 Jun 9;14(6):e0008378. doi: 10.1371/journal.pntd.0008378 (PMC7282628; doi:10.1371/journal.pntd.0008378)
Supplement: S7 Table — * = Unanswered questions were discarded in the statistical analysis ** = High School/Undergraduate Degree *** = Elementary and Middle School **** = Amount equivalent to a minimum monthly salary in Brazil, on 11/31/2016, according the Brazilian Central Bank rc = reference category. (PDF) [file pntd.0008378.s007.pdf]

S7_Table

S7 Table – Univariate analysis of factors potentially associated with helminth infections in dogs from the 10 districts of the Municipality of Ilhéus, Bahia, Brazil (n=143)*.

Variable		n	infected (%)	p-value	OR	95% CI
Age	< 1 year	53	39 (73.6)	-	rc	-
	> 1 year	90	58 (64.4)	0.26	0.65	0.30-1.37
Sex	Female	59	42 (71.2)	-	rc	-
	Male	84	55 (65.5)	0.47	0.76	0.37-1.58
Level of restriction	Restricted	37	27 (72.9)	-	rc	-
	Semirestricted	106	70 (66.0)	0.44	0.72	0.31-1.65
Breed	Yes	30	17 (56.7)	-	rc	-
	No	113	80 (70.8)	0.14	1.85	0.81-4.24
Local	Semirural	41	26 (63.4)	-	rc	-
	Rural	102	71 (69.6)	0.47	1.32	0.62-2.83
Level of education of dog owners	HS/Undergraduated**	30	20 (66.7)	-	rc	-
	E/M School***	98	68 (69.4)	0.78	1.13	0.47-2.71
Income level	> US$ 258.82****	35	19 (54.3)	-	rc	-
	≤ US$ 258.82	97	70 (72.2)	0.06	2.18	0.98-4.85
Contact with other dogs	No	30	21 (70)	-	rc	-
	Yes	113	76 (67.3)	0.77	0.88	0.36-2.11
Exposure to untreated water	No	18	10 (55.5)	-	rc	-
	Yes	122	84 (68.8)	0.27	1.77	0.64-4.83
Anthelminthic treatment	Yes	112	71 (63.4)	-	rc	-
	No	30	25 (83.3)	0.04	2.88	1.03-8.12

*= Unanswered questions were discarded in the statistical analysis
**= High School/Undergraduate Degree
***= Elementary and Middle School
****= Amount equivalent to a minimum monthly salary in Brazil, on 11/31/2016, according the Brazilian Central Bank
rc = reference category
