# Supplementary material for: Enteric parasitic infections in children and dogs in resource-poor communities in northeastern Brazil: Identifying priority prevention and control areas
Source: PLoS Negl Trop Dis. 2020 Jun 9;14(6):e0008378. doi: 10.1371/journal.pntd.0008378 (PMC7282628; doi:10.1371/journal.pntd.0008378)
Supplement: S8 Table — * = Unanswered questions were discarded in the statistical analysis ** = High School/Undergraduate Degree *** = Elementary and Middle School **** = Amount equivalent to a minimum monthly salary in Brazil, on 11/31/2016, according the Brazilian Central Bank rc = reference category. (PDF) [file pntd.0008378.s008.pdf]

S8_Table

S8 Table – Univariate analysis of factors potentially associated with protozoa infections in dogs from the 10 districts of the Municipality of Ilhéus, Bahia, Brazil (n=143)*.

Variable		n	Infected (%)	p-value	OR	95% CI
Age	< 1 year	53	21 (39.6)	-	rc	-
	> 1 year	90	30 (33.3)	0.45	0.76	0.38-1.54
Sex	Female	59	27 (45.8)	-	rc	-
	Male	84	24 (28.6)	0.04	0.47	0.24-0.95
Level of restriction	Restricted	37	9 (24.3)	-	rc	-
	Semirestricted	106	42 (39.6)	0.10	2.04	0.87-4.75
Breed	Yes	30	9 (30)	-	rc	-
	No	113	42 (37.2)	0.47	1.38	0.58-3.29
Local	Semirural	41	13 (31.7)	-	rc	-
	Rural	102	38 (37.2)	0.53	1.28	0.59-2.76
Level of education of dog owners	HS/Undergraduated**	30	14 (46.7)	-	rc	-
	E/M School***	98	35 (35.7)	0.28	0.64	0.28-1.45
Income level	> US$ 258.82****	35	12 (34.3)	-	rc	-
	≤ US$ 258.82	97	37 (38.1)	0.69	1.18	0.52-2.65
Contact with other dogs	No	30	9 (30)	-	rc	-
	Yes	113	42 (37.2)	0.47	1.38	0.58-3.29
Exposure to untreated water	No	18	7 (38.9)	-	rc	-
	Yes	122	44 (36.1)	0.82	0.88	0.32-2.45
Anthelminthic treatment	Yes	112	38 (33.9)	-	rc	-
	No	30	13 (43.3)	0.34	1.49	0.65-3.38

*= Unanswered questions were discarded in the statistical analysis
**= High School/Undergraduate Degree
***= Elementary and Middle School
****= Amount equivalent to a minimum monthly salary in Brazil, on 11/31/2016, according the Brazilian Central Bank
rc = reference category
