# Supplementary material for: Enteric parasitic infections in children and dogs in resource-poor communities in northeastern Brazil: Identifying priority prevention and control areas
Source: PLoS Negl Trop Dis. 2020 Jun 9;14(6):e0008378. doi: 10.1371/journal.pntd.0008378 (PMC7282628; doi:10.1371/journal.pntd.0008378)
Supplement: S9 Table — * = Unanswered questions were discarded in the statistical analysis ** = High School/Undergraduate Degree *** = Elementary and Middle School **** = Amount equivalent to a minimum monthly salary in Brazil, on 11/31/2016, according the Brazilian Central Bank rc = reference category. (PDF) [file pntd.0008378.s009.pdf]

S9_Table

S9 Table – Bivariate analysis of factors potentially associated with coinfections in dogs from the 10 districts of the Municipality of Ilhéus, Bahia, Brazil (n=143)*.

Variable		n	Infected (%)	p-value	OR	95% CI
Age	< 1 year	53	22 (41.5)	-	rc	-
	> 1 year	90	28 (31.1)	0.21	0.64	0.31-1.29
Sex	Female	59	26 (44.1)	-	rc	-
	Male	84	24 (28.6)	0.06	0.51	0.25-1.02
Level of restriction	Restricted	37	9 (24.3)	-	rc	-
	Semirestricted	106	41 (38.7)	0.11	1.96	0.84-4.58
Breed	Yes	30	6 (20)	-	rc	-
	No	113	44 (38.9)	0.06	2.55	0.96-6.74
Local	Semirural	41	12 (29.3)	-	rc	-
	Rural	102	38 (37.2)	0.37	1.43	0.65-3.14
Level of education of dog owners	HS/Undergraduated**	30	13 (43.3)	-	rc	-
	E/M School***	98	37 (37.7)	0.58	0.79	0.34-1.81
Income level	> US$ 258.82****	35	12 (34.3)	-	rc	-
	≤ US$ 258.82	97	37 (38.1)	0.69	1.18	0.52-2.65
Contact with other dogs	No	30	8 (26.7)	-	rc	-
	Yes	113	42 (37.2)	0.29	1.62	0.66-3.98
Exposure to untreated water	No	18	6 (33.3)	-	rc	-
	Yes	122	43 (35.2)	0.87	1.10	0.38-3.10
Anthelminthic treatment	Yes	112	36 (32.1)	-	rc	-
	No	30	14 (46.7)	0.14	1.84	0.81-4.19

*= Unanswered questions were discarded in the statistical analysis
**= High School/Undergraduate Degree
***= Elementary and Middle School
****= Amount equivalent to a minimum monthly salary in Brazil, on 11/31/2016, according the Brazilian Central Bank
rc = reference category
